# Non-communicable disease care and management in two sites of the Cape Town Metro during the first wave of COVID-19: A rapid appraisal

**DOI:** 10.4102/phcfm.v14i1.3215

**Published:** 2022-01-18

**Authors:** Peter A. Delobelle, Mumtaz Abbas, Ishaaq Datay, Angela De Sa, Naomi Levitt, Darcelle Schouw, Steve Reid

**Affiliations:** 1 Department of Medicine, Faculty of Health Sciences, University of Cape Town, Cape Town, South Africa; 2 Department of Public Health, Faculty of Medicine and Pharmacy, Vrije Universiteit Brussel, Brussels, Belgium; 3 Department of Health, Western Cape Government, Cape Town, South Africa; 4 Primary Health Care Directorate, Faculty of Health Sciences, University of Cape Town, Cape Town, South Africa; 5 Department of Family and Emergency Medicine, Faculty of Medicine and Health Sciences, Stellenbosch University, Cape Town, South Africa

**Keywords:** non-communicable diseases, community-orientated primary care, covid-19, service reorganisation, community health workers, type-2 diabetes, rapid appraisal

## Abstract

**Background:**

Non-communicable diseases (NCDs), including type-2 diabetes and hypertension, have been associated with increased morbidity and mortality rates because of coronavirus disease 2019 (COVID-19). Maintaining quality care for these conditions is important but data on the impact of COVID-19 on NCD care in South Africa are sparse.

**Aim:**

This study aimed to assess the impact of COVID-19 on facility and community-based NCD care and management during the first COVID-19 wave.

**Setting:**

Two public health sector primary care sites in the Cape Town Metro, including a Community Orientated Primary Care (COPC) learning site.

**Methods:**

A rapid appraisal with convergent mixed-methods design, including semi-structured interviews with facility and community health workers (CHWs) (*n* = 20) and patients living with NCDs (*n* = 8), was used. Interviews were conducted in English and Afrikaans by qualified interviewers. Transcripts were analysed by thematic content analysis. Quantitative data of health facility attendance, chronic dispensing unit (CDU) prescriptions and routine diabetes control were sourced from the Provincial Health Data Centre and analysed descriptively.

**Results:**

Qualitative analysis revealed three themes: disruption (cancellation of services, fear of infection, stress and anxiety), service reorganisation (communication, home delivery of medication, CHW scope of work, risk stratification and change management) and outcomes (workload and morale, stigma, appreciation and impact on NCD control). There was a drop in primary care attendance and an increase in CDU prescriptions and uncontrolled diabetes.

**Conclusion:**

This study described the service disruption together with rapid reorganisation and change management at primary care level during the first COVID-19 wave. The changes were strengthened by the COPC foundation in one of the study sites. The impact of COVID-19 on primary-level NCD care and management requires more investigation.

## Introduction

In South Africa, there is a significant unmet need for care for non-communicable diseases (NCDs), which already account for nearly 60% of all deaths.^1^ It is estimated that 4 million South Africans have diabetes and 18 million have hypertension, with high rates of both groups being undiagnosed and uncontrolled. For example, close to 70% of people diagnosed with diabetes and 90% of those diagnosed with hypertension are uncontrolled.^2^

When the first coronavirus disease 2019 (COVID-19) wave hit South Africa in March 2020, it was found that older adults and patients with comorbidities were more likely to suffer from severe disease and death.^3^ Amongst these comorbidities, NCDs, in particular diabetes and hypertension, were reportedly associated with a poor COVID-19 prognosis.^4^ Maintaining quality care and treatment of people with these conditions was hence regarded as critical.

The lockdown measures for tackling COVID-19 introduced to reduce transmission of the virus potentially impacted NCDs in several ways, including attendance to care and self-management; however, little data were available to support this hypothesis. Physical distancing and self-isolation were also found to affect NCD risk factor management, for example, through increased tobacco and alcohol use, and its impact on mental health,^5^ although a total ban on the sale of tobacco and alcohol was imposed during the first wave. In addition, delaying routine medical appointments and laboratory tests were feared to have a negative influence on NCD management and result in higher morbidity, disability and avoidable mortality.

This rapid appraisal study aimed to assess the impact of the first COVID-19 wave on the management and care of NCD patients at primary care level in the Cape Town Metro. Two primary care sites were selected, one of which had been a Community Orientated Primary Care (COPC) learning site since 2017. The COPC strategy entails close liaison with community outreach teams consisting of community health workers (CHWs) employed by a local non-profit organisation (NPO) as described elsewhere.^6^ This primary health care (PHC) strategy was reinforced at the start of the first COVID-19 wave, at both facility and community level.^7^

## Methods

### Study design

A rapid appraisal design with convergent mixed methods was used to provide information to decision makers in a timely and cost-effective way.^8^ Rapid appraisal has been used to assess aspects of PHC in South Africa and elsewhere and emphasises community participation.^8^

### Setting

This study was conducted in two primary care sites in the public sector in the Cape Town Metro, including one COPC learning site. Each site serves a population of around 60 000 mostly mixed-race English and Afrikaans speaking inhabitants.

### Study population and data collection

Data were collected using semi-structured interviews with facility managers and professional healthcare workers in both sites involved in NCD care and management (*n* = 9), with CHWs, professional nurses and managers from the NPOs employed in their catchment areas (*n* = 11) and with patients living with NCDs, specifically type-2 diabetes and hypertension (*n* = 8) (Table 1). Participants were purposively sampled and invited to participate after verbal and written informed consent was obtained. Interviews were limited in time to avoid interfering with the workload caused by COVID-19 for health workers and conducted telephonically or via Zoom in line with COVID-19 restrictions.

**TABLE 1:** Study participants interviewed per site.

Variable	Site A (COPC learning site)	*n*	Site B	*n*
Facility workers	Facility manager	1	Facility manager	1
	Senior family physician	1	Senior family physician	1
	Clinical nurse practitioners	4	Clinical program coordinator	1
Community-based services	Director	1	Coordinator	1
	Programme manager	1	Project manager	1
	Professional nurse	1	Professional nurses	2
	Community health worker	1	Community health workers	3
Patients	Clients	3	Clients	5

COPC, community orientated primary care.

The interviews were conducted using a semi-structured topic guide developed in English and translated into Afrikaans and conducted in the language of choice of participants. Interviews were conducted by English and Afrikaans speaking researchers qualified in qualitative data collection, digitally recorded and transcribed verbatim. All data were collected in the period October 2020 – November 2020 between the first and second wave of COVID-19.

### Data analysis

Transcripts were analysed using open coding to identify themes in line with study objectives. In addition, an inductive process was used to identify emerging themes related to the topic and identified by constant comparison until saturation was reached. The analysis was conducted independently by two researchers and supported by qualitative analysis software (Atlas.ti version 8).

Secondary data were collected regarding health facility attendance, chronic dispensing unit (CDU) prescriptions and routine assessment of diabetes control. Aggregated data for the period 2019–2020 were obtained from the Provincial Health Data Centre, analysed descriptively and used to triangulate study’s findings.

### Ethical considerations

Ethics approval was obtained from the Human Research Ethics Committee of the University of Cape Town (reference: 307/2020) and permission to conduct the study obtained from the Western Cape Government Department of Health (reference: WC_202008_075). Confidentiality of all study participants was maintained throughout data collection and analysis.

## Results

### Qualitative findings

**FIGURE 1:**
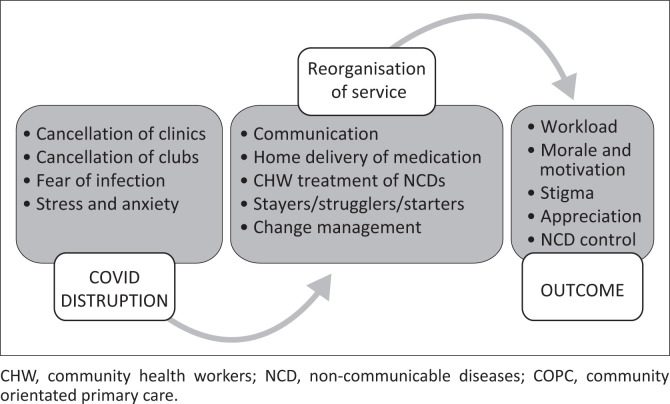
Themes identified from interview transcripts (*n* = 28).

Thematic content analysis of the interview transcripts revealed several categories that were grouped in three chronologically sequential themes related to the impact of COVID-19, situated within a context of local social determinants of health and COPC roll-out (Figure 1). The categories included factors related to COVID-19 disruption, service reorganisation and related outcomes. The disruption (cancellation of clinics and clubs, fear of infection amongst health workers and patients, stress and anxiety) resulted in services being reorganised, characterised by improved communication between health workers and patients, home delivery of medication, changed CHW scope of work and risk stratification of NCD patients, which required active change management. The disruption and reorganisation affected the workload, morale and motivation of health workers, resulting not only in (secondary) stigma because of health workers’ association with COVID-19 but also in appreciation for services delivery, all of which impacted NCD control.

### COVID-19 disruption

At the onset of the first wave, routine clinic services and ‘chronic clubs’ for NCDs patients were suddenly cancelled. This was confusing for staff and patients alike, and the high level of uncertainty caused much stress and anxiety, as many high-risk patients did not visit the facility for fear of infection:

‘It obviously had a profound effect on the services that we were able to offer. I have a grave concern that a lot of our chronic patients have sort of like disappeared.’ (B2, medical officer, female)

Facility staff also feared infection with COVID-19 themselves and, in some cases, refused to work. Staff members, and especially those with comorbidities, were afraid when another staff member tested positive. Those who showed symptoms of COVID-19 and were tested positive spread fear of infection amongst their colleagues:

‘There was a lot of pressure, and we were very, very scared, especially if at times we found out that one of our members tested positive.’ (A5, clinical nurse practitioner, female)

Community health workers also feared infection with COVID-19, especially as they were required to deliver medication at patients’ homes. They feared infecting their own family members, those with comorbidities and the elderly. They also faced threats when delivering medication, and in some instances, even faced stone throwing by the local community, who viewed the CHWs as carriers of COVID-19. Some patients were particularly apprehensive in the beginning of lockdown because of the fear of the virus being passed onto them:

‘For me it was a bit stressful because of going out to the clients, you have to go back to your house, you can get the virus, you can take it back home, but in the end I just did what I had to do and provide support for the family.’ (A10, community health worker, female)

### Reorganisation of services

The cancellation of clinics and clubs resulted in a new system of booking being introduced, whereby patients were allocated time slots and only seen at that time. This enabled the clinics to be decongested. In Site A, patients were categorised into ‘stayers’ (those under control), ‘strugglers’ (uncontrolled NCD/defaulters), ‘starters’ (newly diagnosed) and ‘walk-ins’. Stayers would, unless they presented new problems, get a repeat script for six months. One month before their scheduled in-person visit, they would attend for blood tests, whilst medication was delivered at home for the months in between. Strugglers were encouraged to visit the facility, where they consulted with the doctor, dietician or health promoter as often as needed to achieve control:

‘We’re first going to quickly sort out the stayers so that they can go, then we sort out our strugglers, see if we must adjust your medication, what is the reason why is your blood pressure always high, refer you to the dietician.’ (A4, clinical nurse practitioner, female)

In Site A, professional nurses, medical officers, pharmacists and CHWs discussed challenges they were experiencing and aimed to resolve it within the COPC or staff WhatsApp groups. A dedicated appointment line was also established, whereby patients were able to secure an appointment with a healthcare provider by sending a text message, WhatsApp or ‘please call me’ using the appointment line:

‘So the patients can WhatsApp for an appointment, they can send a please call me, they can send a text message or they can phone on this line and someone will respond to them within 30 min.’ (A2, medical officer, female)

New booking systems were also tested to ensure efficient handling of patient numbers. Mid-way through the first COVID-19 wave, through trial-and-error, systems had been put in place, which ensured that patients received their medication. The system required efficient teamwork and communication, despite there being a shortage of staff:

‘[*There*] were a lot of adjustments, it was like trial and error. There was no book that told us how to do things; it was literally just trial and error.’ (A1, facility manager, female)

Home delivery of medication was motivated by the urgent need to decongest health centres and protect people from increased exposure to COVID-19. Having a CHW platform enabled home delivery of medication, but there was no process or procedure in place to direct how the service should be run. Many challenges were faced with incorrect addresses, out of area patients, Uber challenges and stock outs. Patients’ addresses were however sorted via direct communication between patients and healthcare workers:

‘We would have their medication, but our patients move around a lot. So sometimes we can’t get hold of them on the telephone or they don’t live at that address anymore and they didn’t inform us.’ (A2, medical officer, female)

Community health workers were also instrumental in checking up on NCD patients during their home visits and feeding the information to the professional nurse. This information was valuable to identify which patient was receiving medication in order to ensure adherence and compliance with their medication. Community health workers assisted chronic patients by counting the remaining medication at their homes and encouraging them to take their medication regularly:

‘I do pill counting with my chronic patients, [if they] have not used their medication yet, then I encourage the person or I will see that the person [*does so*]. When I have done with the person I need to do, wash, whatever, then will I see the person, I will talk to the family member can the person please …’ (B9, community health worker, female)

The reorganisation of services, therefore, entailed an increased role for CHWs (task shifting), who ensured that medication, patient screening, addresses and clinic visits were organised in a system to minimise the risks and impact of COVID-19. Facility staff worked closely with CHWs to shape how the service was delivered, resulting in improved relationships to ensure rapid service implementation:

‘We wouldn’t have been able to do it without them because with them they have now taken the medication to the people … And as I say we don’t think of each other as a separate entity I’m thinking of the NPO manager as one of my staff members.’ (A1, facility manager, female)

### Outcomes

At the start of lockdown, one of the sites was extremely busy with COVID-19 testing, which resulted in many positive cases that overwhelmed the capacity of laboratory staff. Staff shortage was reported as adding to the workload. Morale of staff was initially low because of working in high-risk environments which was exacerbated when staff became ill as a result of COVID-19 and when they had to care for ill family members or when they simply refused to work:

‘Morale was very low because everyone was fearful and everyone worked in the COVID-19 unit where they were exposed to suspected cases.’ (B2, medical Officer, female)

As services were reorganised and new systems for home delivery of medication and patient care put in place, workload reduced gradually and staff were able to work more efficiently. Facilities also implemented strategies and processes to streamline services to reduce the risk of infection and accommodate unstable patients, which relieved the pressure on staff. Community health workers, however, experienced an increased workload, as they worked longer hours and walked long distances carrying heavy loads of medication, although they did it with love and compassion:

‘So, for me it was, I did it out of love. My work, I have a love for my work, for the community. I care. I’m going to try to help where I can, I’m going to refer to where I can refer.’ (B8, community health worker, female)

At the start of COVID-19, there was also stigma attached to the disease, which was reduced as more people were educated and gained a better understanding of COVID-19. Stigma was attached to staff members who were thought to be responsible for contracting the virus, and CHWs were stigmatised for being associated with the disease when delivering medication. People sometimes locked their doors for fear of infection, but also started to appreciate the home delivery of medication, as they were able to save money, avoid risk of infection and avoid the inconvenience of standing in long queues to collect medication:

‘The people really appreciate what we do about the chronic dispensing units [*CDU*], because they save money, they save for the day hospital, as we do them.’ (A10, community health worker, female)

Non-communicable disease control was, however, difficult to monitor because of the lack of efficient systems for dealing with unstable patients prior to COVID-19 and defaulters were not easily traced. Because of COVID-19, many patients were unemployed, hence they could not buy food and medication consistently and control their chronic disease:

‘We’ve seen a lot of uncontrolled patients, come in very sick … and I think it’s also because a lot of people have lost their jobs, they’re not eating well.’ (B2, medical officer, female)

### Quantitative results

Aggregated data sourced from the Provincial Research Data Centre and study sites in terms of PHC headcount, CDU prescriptions, number of patients with diabetes and hemoglobin A1c (HbA1c) tests, revealed a sharp decrease in the number of patients visiting their facility in 2020 compared with 2019, accompanied by an increase in the number of CDU prescriptions. The number of HbA1c tests fell sharply in proportion to the drop in visits, and the proportion of people with uncontrolled diabetes was proportionally higher in 2020 compared with 2019 at both sites (Table 2). The difference in change was less pronounced in the COPC site (Site A) compared with the other study site.

**TABLE 2:** Comparison of health indicators for study facilities, 2019–2020.

	Site A (COPC)					Site B				
	2019		2020		Δ%	2019		2020		Δ%
	*n*	%	*n*	%		*n*	%	*n*	%	
PHC headcount	193 839	-	131 071	-	−32	282 262	-	147 209	-	−48
CDU prescriptions	17 969	-	34 679	-	+93	49 094	-	76 571	-	+56
Diabetes patients	2047	-	2067	-	+1	3602	-	3596	-	0
HbA1c tests	1154	-	617	-	−47	2269	-	933	-	−59
Normal < 6	82	7	42	7	0	168	7	65	7	0
Controlled 6–8	362	31	168	27	−4	743	33	258	28	−5
Poor control 8–10	335	29	163	26	−3	661	29	218	23	−6
Uncontrolled > 10	375	32	244	40	+8	697	31	392	42	+11

*Source:* Provincial Health Data Centre, Western Cape Government.

COPC, community orientated primary Care; PHC, primary health care; CDU, chronic dispensing unit; HbA1c, hemoglobin A1c.

## Discussion

This rapid appraisal of the impact of COVID-19 on NCD services delivery in two primary care sites in the Cape Town Metro highlighted the disruption of routine clinical services, alongside their reorganisation, including home-based delivery of medication and improved communication amongst healthcare workers and patients. COVID-19 also instilled fear and anxiety amongst healthcare workers and patients. COVID-19 not only impacted the workload, morale and motivation of health workers, accompanied by stigma, but also appreciation for the continued provision of essential services and ongoing efforts to control NCDs.

The disruption of clinical services was in line with projections in anticipated health service demands in the Western Cape, which in March – April 2020 focused on speedily de-escalating and adapting routine services.^7^ The latter included deferring NCDs and rehabilitation visits and elective services, whilst offering home delivery of medication for repeat prescriptions, booking patients according to specific appointment times and dissuading those with minor ailments from attending facilities.^8^ Some clinics were closed for short periods to allow for decontamination following confirmation of COVID-19 cases; staff absence because of illness or redeployments to COVID-19 services or fear amongst patients of contracting the virus during follow-up visits.^9,10^

Routine data from the District Health Information System in 2019–2020 indicated restricted access to public health services under strict lockdown regulations, with the largest decline reported in the Western Cape.^10^ A study looking at the impact of COVID-19 on the number of specimens sent to the Tygerberg public sector laboratories showed a significant decline in the number of tests for NCDs (including 59% reduction in tests for lipids and 64% for creatinine and HbA1c) between March and June 2020 compared with the same period in 2019.^9^ In this study, we also found a sharp decrease in the number of HbA1c tests carried out in 2020 compared with 2019.

Fear of infection amongst healthcare workers at the onset of the first wave was common and highlighted in the media with increasing numbers of health workers infected with and dying of COVID-19.^11,12^ A survey from the Human Sciences Research Council and the University of Kwazulu-Natal indicated that around a fifth of healthcare workers surveyed in the period April 2020 – May 2020 were severely distressed, particularly nurses and those working in the public sector.^13^ Results revealed a major concern for personal and family well-being and passing COVID-19 infection to family members, especially amongst nurse practitioners.^13^ A survey conducted in July 2020 – August 2020 amongst primary care nurses in the Western Cape also pointed to the need for comprehensive support to manage stress and anxiety.^14^

The disruption caused by COVID-19 led to a major reorganisation of primary care services, guided by directives for PHC preparedness under COVID-19,^15^ which had both negative and positive effects on NCDs. Limited access to clinic services resulted in patients with NCDs not seeking help because of fear of infection with COVID-19. This was mitigated to some extent by home delivery of medication, which took off rapidly and was reported to be successful in the Cape Town Metro.^16^ Home delivery of medication also helped to decongest facilities, as shown elsewhere,^17^ and to prevent spread amongst those patients with comorbidities who were at higher risk of severe disease. However, incorrect addresses and telephone numbers proved problematic for the home delivery system.

Especially in the COPC site, where previous work had focused on streamlining facility- and community-based services, this service reorganisation occurred more rapidly, as suggested elsewhere.^8^ Before the introduction of COPC, non-adherence to diabetes care in this site was found to be linked to personal and structural barriers, such as overburdened facilities, poor support structures, safety concerns and low income and unemployment.^18^ In the other site, the close working relationship between facility staff and CHWs also prompted an effective response, but there was a larger decrease in the number of HbA1c tests performed and relatively more uncontrolled patients.

This study revealed improved communication between facility and community-based health workers, including around the coordination of home delivery of medication.^16^ In the first month of lockdown, CHWs delivered 184 000 parcels to households.^8^ Community health workers also conducted community screening and testing in vulnerable communities,^16^ as well as COVID-19-related health education and risk communication. These tasks were added to their routine work, such as home-based care, tuberculosis (TB) and human immunodeficiency virus (HIV) follow-up and defaulter tracing and could be perceived as appropriate because well controlled lower risk patients were monitored by CHWs, whilst those at higher risk were selected for review. Non-communicable diseases risk stratification and differentiation of level of care related to reorganisation of services because of COVID-19 could hence be seen as a positive result, or so-called ‘COVID-19 blessing’. Retention in care of patients on chronic medication could also potentially be tightened as information systems make tracking more feasible.

Finally, in this study, COVID-19 was found to increase the workload of facility healthcare workers at the start of the pandemic. Many health workers were put under strain, affecting their morale and motivation, as anticipated.^19^ This changed as facilities became decongested and healthcare workers were more knowledgeable about COVID-19, in turn relieving and improving health worker morale and motivation. The initial stigma was also reported to diminish and be replaced by a sense of appreciation for the efforts of healthcare workers, in particular CHWs, to continue delivering services, despite their fear of infection and initial stigmatisation.

The overall impact on NCD care and management was however difficult to assess, as the net improvement or deterioration of NCDs control in the study period was hard to measure. The proportion of uncontrolled HbA1c readings seemed to be higher in 2020 compared with 2019, which could be partly explained by a tighter risk stratification, as only those at higher risk (‘strugglers’) were requested to visit the facility for medical review and their blood test was carried out. The results should also be interpreted in view of delays in help-seeking behaviour because of the reluctance of patients to attend clinics for fear of infection. It is worth noting, however, that the systems put in place in the first COVID-19 wave benefitted from additional funding to ensure home delivery of medication, which should be taken into account when planning sustainable health policy and management strategies in the post-COVID era.

Although this study was limited to two urban sites and used a purposive sample of healthcare workers and patients, which may have introduced selection bias, the findings were triangulated by sources of data and by methods, and hence, offer credible insights into the strengths and limitations of service reorganisation on the management and care of NCDs during COVID-19. No substantial difference could be found on the impact of the first wave of COVID-19 on NCD service indicators between both sites, but the COPC foundation in one site appeared to provide a stronger basis on which to re-organise services in a coordinated manner.

## Conclusion

This study in two primary care sites in the Cape Town Metro has highlighted the impact of COVID-19 on NCD services disruption and reorganisation and its impact on facility and community-based health workers and patients. The findings offer some indication as to how NCD health policy could be informed post-COVID-19, for example, by retaining the home delivery of medication. Community orientated primary care foundations in one site facilitated a rapid and efficient service re-organisation, using risk stratification and tight communication between facility, CHWs and patients. Home delivery of medication was highly appreciated; however, more research studies are needed to measure the actual impact of COVID-19 on NCDs care and management.
