# Corrigendum: Non-communicable disease care and management in two sites of the Cape Town Metro during the first wave of COVID-19: A rapid appraisal

**DOI:** 10.4102/phcfm.v14i1.3443

**Published:** 2022-09-30

**Authors:** Peter A. Delobelle, Mumtaz Abbas, Ishaaq Datay, Angela De Sa, Naomi Levitt, Darcelle Schouw, Steve Reid

**Affiliations:** 1 Department of Medicine, Faculty of Health Sciences, University of Cape Town, Cape Town, South Africa; 2 Department of Public Health, Faculty of Medicine and Pharmacy, Vrije Universiteit Brussel, Brussels, Belgium; 3 Department of Health, Western Cape Government, Cape Town, South Africa; 4 Primary Health Care Directorate, Faculty of Health Sciences, University of Cape Town, Cape Town, South Africa; 5 Division of Family Medicine, School of Public Health and Family Medicine, Faculty of Health Sciences, University of Cape Town, Cape Town, South Africa; 6 Department of Family and Emergency Medicine, Faculty of Medicine and Health Sciences, Stellenbosch University, Cape Town, South Africa

In the published article, Delobelle AP, Abbas M, Datay I, et al. Non-communicable disease care and management in two sites of the Cape Town Metro during the first wave of COVID-19: A rapid appraisal. Afr J Prm Health Care Fam Med. 2022;14(1), a3215. https://doi.org/10.4102/phcfm.v14i1.3215, there was an error in affiliation 1 for co-author 4, Angela De Sa. Instead of ‘Department of Medicine, Faculty of Health Sciences, University of Cape Town, Cape Town, South Africa’, it should be ‘Division of Family Medicine, School of Public Health and Family Medicine, Faculty of Health Sciences, University of Cape Town, Cape Town, South Africa’.

The authors apologise for this error. The correction does not change the significance of study’s findings or overall interpretation of its results or the scientific conclusions of the article in any way.

